# Gut Microbiome and Metabolome Signatures Associated with Heat Tolerance in Dairy Cows

**DOI:** 10.3390/microorganisms13122829

**Published:** 2025-12-12

**Authors:** Mingxun Li, Peng Chen, Can Liu, Shimeng Wang, Hao Zhang, Jiaxi Li, Niel A. Karrow, Yongjiang Mao, Zhangping Yang

**Affiliations:** 1 Key Laboratory of Animal Genetics & Breeding and Molecular Design of Jiangsu Province, College of Animal Science and Technology, Yangzhou University, Yangzhou 225009, China; limingxun@live.com (M.L.); mx120240895@stu.yzu.edu.cn (P.C.); lc1714543081@163.com (C.L.); mz120241629@stu.yzu.edu.cn (S.W.); mx120250916@stu.yzu.edu.cn (H.Z.); mx120250941@stu.yzu.edu.cn (J.L.); cattle@yzu.edu.cn (Y.M.); 2 Center for Genetic Improvement of Livestock, Department of Animal Biosciences, University of Guelph, Guelph, ON N1G 2W1, Canada; nkarrow@uoguelph.ca

**Keywords:** holstein cows, heat stress, intestinal microbiota, metabolomics, biomarkers

## Abstract

Heat stress significantly impairs dairy cow health and productivity, highlighting the need to understand the gut microbiome–metabolite interactions that contribute to heat tolerance. Here, we integrated metagenomic sequencing and untargeted metabolomics in twelve holstein cows selected from a previously phenotyped herd of 120 individuals, including six heat-tolerant (HT) and six heat-sensitive (HS) cows identified using entropy-weighted TOPSIS scoring. HT cows were enriched in genera such as *Faecalimonas* and *UBA737*, which were functionally linked to pathways of energy and lipid metabolism, whereas, HS cows harbored taxa associated with bacterial lipopolysaccharide and glycosphingolipid biosynthesis. A total of 135 metabolites were differentially abundant between groups. Among them, glycerol 2-phosphate and 24(28)-dehydroergosterol showed perfect classification performance (AUC = 1.000), and were mainly involved in membrane lipid remodeling and redox regulation. Integrated analysis revealed coordinated microbial–metabolite networks, exemplified by the *Faecalimonas*–LysoPS (16:0/0:0) and *UBA737*–Glycerol 2-phosphate axes, suggesting functional coupling between microbial composition and metabolic adaptation. Together, these findings demonstrate that HT cows harbor gut microbiota and metabolites favoring energy balance, membrane remodeling, and oxidative stress resilience, while HS cows display stress-related metabolic patterns. This study elucidates the microbial–metabolic mechanisms underlying thermal resilience and highlights potential biomarkers and metabolic pathways that could be applied in heat-tolerance breeding and precision management of dairy cattle.

## 1. Introduction

Heat stress represents a major environmental threat to global dairy production, particularly in high-yielding Holstein cows that exhibit limited thermoregulatory capacity due to intensive metabolic heat generation [1]. In dairy cattle, heat stress typically occurs when the temperature–humidity index (THI) exceeds 68, corresponding to ambient temperatures of 26–32 °C and relative humidity above 50% [2]. Even 4–6 h of daily exposure to such conditions can disrupt physiological homeostasis, leading to a 10–25% decline in milk yield and estimated global economic losses exceeding one billion USD annually [1,3]. Exposure to excessive ambient temperatures disrupts endocrine and immune homeostasis, induces oxidative stress, and reduces feed intake, ultimately impairing milk yield, fertility, and animal welfare [4,5]. With climate change increasing the frequency and intensity of heat events, enhancing the natural heat tolerance of dairy cattle has become a major priority for sustainable production systems.

Emerging evidence has highlighted a pivotal role of the gut microbiota in mediating host adaptation to heat stress. The intestinal microbiota regulates nutrient absorption, energy metabolism, and immune responses, thereby serving as a key factor in maintaining physiological homeostasis under environmental challenges [6,7]. Heat stress can disrupt this microbial ecosystem, leading to compositional shifts in dominant taxa such as *Bacteroidota*, *Firmicutes*, and *Verrucomicrobiota*, which in turn may compromise intestinal barrier function and metabolic efficiency [8,9,10]. These microbial alterations are frequently accompanied by reduced short-chain fatty acid (SCFA) production, impaired mucosal barrier integrity and increased endotoxin translocation into the circulation, ultimately contributing to systemic inflammation and oxidative imbalance in the host [11,12]. Consistent with these observations, our previous rumen metagenomic study demonstrated marked differences in microbial composition and metabolic pathways between heat-tolerant (HT) and heat-sensitive (HS) cows, with *Bacteroidota*, *Firmicutes* and *Spirochaetota* contributing to distinct adaptive strategies [13]. However, despite accumulating evidence on microbial adaptations to environmental stress, the specific contribution of intestinal microbial–metabolite interactions to thermal resilience in dairy cows has not been systematically characterized.

Although recent studies have advanced our understanding of the intestinal microbiota under heat stress, most have primarily addressed community composition, while functional and metabolic insights are still emerging. The mechanisms by which gut microbes and their metabolites jointly contribute to thermotolerance have not yet been fully elucidated. Integrating metagenomic sequencing with untargeted metabolomics offers a powerful multi-omics framework to merge microbial functions with downstream metabolic outputs, enabling a systems-level understanding of how the gut microbiota supports physiological adaptation to heat stress [14,15].

Therefore, this study aimed to investigate the gut microbiome and metabolome profiles of HT and HS Holstein cows using integrated metagenomic and untargeted metabolomic analyses. By identifying differential microbial taxa, functional pathways and key metabolites, we sought to elucidate the microbial–metabolite interactions underlying heat tolerance. This work provides novel insights into the role of gut microbial metabolism in thermal adaptation, and identifies potential biomarkers for precision selection and management of heat-resilient dairy cattle.

## 2. Materials and Methods

### 2.1. Animals and Sample Collection

This study was conducted from July to September 2023 at a commercial dairy farm in Suqian, Jiangsu Province, China. All experimental animals were multiparous Holstein cows in mid-lactation, maintained under identical management, housing and feeding conditions. The cows had an average body weight of 640 ± 25 kg and an average daily milk yield of 32.0 ± 2.5 kg during the study period. They were housed in naturally ventilated free-stall barns equipped with high-power fans and automatic misting systems, with a 30-min cooling protocol applied before each milking to reduce heat load.

Based on heat stress responses measured in our previous study [13], twelve cows were selected from a larger population of 120 individuals phenotyped under natural summer heat exposure. For each cow, multiple physiological and production indicators, including rectal temperature, respiratory rate, drooling score, daily milk yield, and behavioral responses, were repeatedly recorded throughout the heat-stress period, together with concurrent monitoring of THI and environmental conditions.

To integrate these multi-dimensional measurements objectively, an entropy-weighted TOPSIS model was applied. The entropy method assigns data-driven weights based on the information content of each indicator, while TOPSIS ranks individuals according to their similarity to an ideal heat-tolerance profile. This method minimizes subjectivity and enables robust identification of contrasting phenotypes. According to their composite TOPSIS scores, the top 5% of cows were classified as heat-tolerant (HT), whereas the bottom 5% were classified as heat-sensitive (HS). Six cows from each extreme group were selected for multi-omics analysis.

Fresh fecal samples (~10 g) were collected rectally between 07:00 and 08:00 a.m. before morning milking using sterile gloves. These samples were immediately snap-frozen in liquid nitrogen, and stored at −80 °C until DNA extraction and metabolomic analysis.

All experimental procedures were approved by the Animal Care and Use Committee of Yangzhou University and conducted in accordance with the Regulation for the Ad-ministration of Affairs Concerning Experimental Animals (Ministry of Science and Technology of China, 2017).

### 2.2. DNA Extraction and Metagenomic Sequencing

Total microbial DNA was extracted from approximately 200 mg of each fecal sample using the QIAamp Fast DNA Stool Mini Kit (Qiagen, Hilden, Germany) following the manufacturer’s protocol. DNA quality and concentration were assessed using a NanoDrop 2000 spectrophotometer (Thermo Fisher Scientific, Waltham, MA, USA) and agarose gel electrophoresis.

Sequencing libraries were prepared using the NEBNext^®^ Ultra™ DNA Library Prep Kit (New England Biolabs, Ipswich, MA, USA) and sequenced on the Illumina NovaSeq 6000 platform (Illumina, San Diego, CA, USA) to generate 150 bp paired-end reads. Raw reads were filtered with Trimmomatic to remove adapters and low-quality base reads [16], and bovine DNA sequences were removed by mapping against the bovine reference genome (*Bos taurus* ARS-UCD1.2, https://www.ncbi.nlm.nih.gov/assembly/GCF_002263795.1, accessed on 8 June 2025) using Bowtie2 [17]. High-quality reads were assembled with MEGAHIT (v1.2.9) [18], and open reading frames (ORFs) were predicted with Prodigal (v2.6.3) [19]. A nonredundant gene catalog was constructed with CD-HIT (v4.8.1) at 95% sequence identity and 90% coverage [20]. Quality-filtered reads were subsequently mapped back to the nonredundant gene catalog with Bowtie2 for quantification, and gene abundances were normalized as transcripts per million (TPM) [17]. Taxonomic annotation was performed using DIAMOND BLASTP against the NCBI NR database (release 2023; E-value < 1 × 10^−5^, identity ≥ 60%) [21], while functional annotation was based on Kyoto Encyclopedia of Genes and Genomes (KEGG, release 2022-12), eggNOG (v5.0), and CAZy databases.

Alpha diversity indices (Shannon, Simpson, ACE, Chao1) and beta diversity (principal coordinates analysis, PCoA) based on Bray–Curtis distances, PERMANOVA) were calculated in R (v4.2.2) [22]. Differential taxa were identified using the Linear discriminant analysis Effect Size (LEfSe) method: the Kruskal–Wallis rank-sum test (α = 0.05) was first applied to detect features with significant differences among groups, followed by pairwise Wilcoxon tests (α = 0.05) for subclass comparisons. Linear discriminant analysis (LDA) was then performed to estimate the effect size with a threshold of LDA score >2.0.

### 2.3. Untargeted Metabolomic Analysis

Untargeted metabolomic profiling of fecal samples was conducted by Shanghai Lingen Biotechnology Co., Ltd., Shanghai, China. Approximately 50 mg of feces was extracted with 400 μL methanol/water (7:3, *v*/*v*), vortexed, and centrifuged at 12,000 rpm for 10 min at 4 °C. The supernatant was analyzed using a UHPLC-Q Exactive Orbitrap MS platform with an HSS T3 column (2.1 × 100 mm, 1.8 μm) under both positive and negative ion modes. One pooled quality control sample was injected after every 10 study samples, and signal normalization and drift correction were based on QC data. Raw data were processed using Compound Discoverer 3.1 for peak extraction, alignment, noise filtering, and normalization. Metabolites were annotated against HMDB (v5.0), KEGG (release 2022-12), and mzCloud databases, and key differential metabolites were further validated with authentic standards.

Principal component analysis (PCA) and partial least squares discriminant analysis (PLS-DA) were performed using SIMCA 14.1 (Umetrics, Umeå, Sweden). Metabolites with VIP ≥ 1.0, |log_2_FC| ≥ 1.0, and *p* < 0.05 were considered significantly different between HT and HS groups. The discriminative ability of potential biomarkers was evaluated by receiver operating characteristic (ROC) curve analysis, which assesses the sensitivity and specificity of classification models. The area under the curve (AUC) values and 95% confidence intervals were calculated using the DeLong method [23,24].

### 2.4. Integrative Analysis of Metagenomic and Metabolomic Data

Associations between differential microbial taxa and metabolites were evaluated by Spearman’s rank correlation analysis using the Hmisc package (v4.8-0) in R (v4.2.2) [25]. Correlations with |*r*| > 0.6 and *p* < 0.05 were considered significant. Clustered heatmaps were generated using the Spearman analysis platform to visualize correlation patterns.

### 2.5. Statistical Analysis

For two-group comparisons, statistical differences in microbial features were evaluated using the Wilcoxon rank-sum test, and those in metabolites were determined by *t*-test. Features with *p* < 0.05 were considered significant.

Given that biological sample sizes of this scale may not always meet normality assumptions, non-parametric statistical approaches (Wilcoxon and LEfSe) were intentionally used for microbiome analyses. Parametric tests were applied to metabolomic data only after verifying distributional assumptions. This statistical framework ensures robust inference while avoiding potential violations of normality in small-sample comparisons.

## 3. Results

### 3.1. Quality Assessment of Macrogenomic Sequencing Data

A total of approximately 105.9 Gb of high-quality metagenomic data were generated, with 8.06–9.32 Gb of clean bases per sample, corresponding to 54.2–62.7 million clean reads. The GC content ranged from 43.9% to 45.7%, with an average of ~45.2%. The sequencing quality was high, with Q20 values above 96.7%, and Q30 values between 91.8% and 94.8% for all samples. After bovine DNA sequence removal, most effective reads were retained. Microbiome assembly yielded high-quality contigs, from which open reading frames were predicted to construct a non-redundant gene catalog comprising several million genes, providing a solid basis for subsequent taxonomic and functional annotations. Detailed sequencing statistics have been provided in Supplementary Table S1.

### 3.2. Gut Microbial Diversity and Community Composition

**Figure 1:**
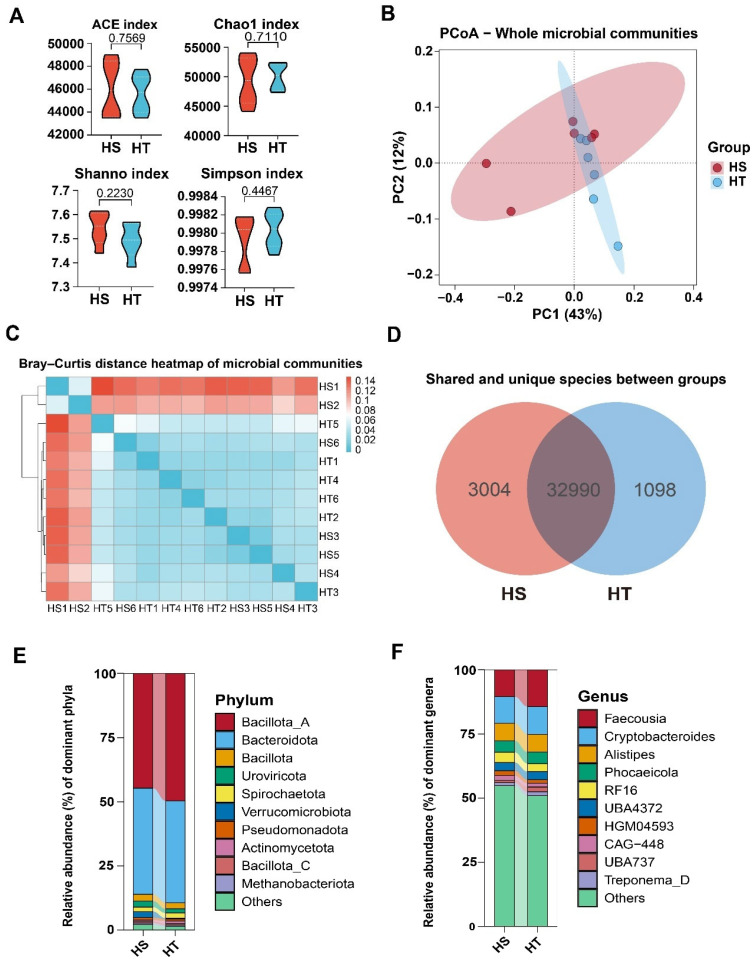
Gut microbial diversity and taxonomic composition in six heat-tolerant (HT) and six heat-sensitive (HS) cows. (**A**) Alpha diversity indices (ACE, Chao1, Shannon, Simpson) calculated from metagenomic sequencing data to evaluate within-sample richness and evenness of gut microbial communities. (**B**) Principal coordinate analysis (PCoA) based on Bray–Curtis distance matrices illustrating differences in overall community structure between HT and HS groups. (**C**) Heatmap of Bray–Curtis distances showing hierarchical clustering of microbial communities, with HT samples forming a more compact cluster than HS samples. (**D**) Venn diagram showing the numbers of shared and unique microbial species between HT and HS cows. (**E**) Relative abundance of dominant bacterial phyla derived from taxonomic profiling of metagenomic reads. (**F**) Relative abundance of dominant bacterial genera, showing the distribution of the most prevalent genera across HT and HS cows.

Analysis of alpha diversity showed no significant differences between HT and HS cows across the ACE, Chao1, Shannon, and Simpson indices (all *p* > 0.2; Figure 1A). The ACE and Chao1 richness estimators were comparable between groups, averaging around 48,000 and 51,000, respectively. The Shannon index showed a slight increase in HS cows, with mean values of 7.6 ± 0.1 compared with 7.5 ± 0.1 in HT cows, whereas the Simpson index was marginally higher in HT cows (0.9984 ± 0.0003 vs. 0.9980 ± 0.0004). These results suggest that overall richness and evenness of the gut microbiota were largely conserved, with only subtle tendencies toward greater diversity in HS and more uniform distribution in HT cows.

In contrast, beta diversity analysis revealed compositional distinctions. The PCoA based on Bray–Curtis distances demonstrated a partial but significant separation between groups (PC1 = 43%, PC2 = 12%), as supported by PERMANOVA (*R*^2^ = 0.1691, *p* = 0.047; Figure 1B). The HT samples clustered more tightly, whereas HS samples showed greater dispersion, indicating that HT cows harbor more structurally homogeneous microbial communities, while HS cows exhibit higher inter-individual variability and potential instability under heat stress.

Furthermore, hierarchical clustering based on Bray–Curtis distances illustrated clear group-level separation, with HT samples forming a more compact cluster compared to the more dispersed HS samples (Figure 1C). This pattern further supports the observation that the microbial communities of HT cows are compositionally more stable and homogeneous, whereas HS cows exhibit greater inter-individual variation under heat stress conditions.

The Venn diagram analysis revealed that the two groups shared a large core microbiota of 32,990 species-level operational taxonomic units (OTUs), while 3004 and 1098 unique taxa were detected exclusively in the HS and HT groups, respectively (Figure 1D). At the phylum level, both groups shared a conserved microbial backbone dominated by *Bacillota_A* and *Bacteroidota*, consistent with their central roles in carbohydrate fermentation and nutrient metabolism in ruminants (Figure 1E). Beyond this overall similarity, *Bacillota_A* accounted for about 56% of the sequences in HT cows, whereas *Bacteroidota* represented roughly 49% in HS cows, indicating a compositional shift in microbial dominance under heat stress. Minor phyla such as *Pseudomonadota* (3–4%), *Spirochaetota* (2–3%), and *Uroviricota* (<2%) were detected in both groups. Notably, *Verrucomicrobiota* was slightly more abundant in HS cows (around 3–4%), a phylum frequently linked to mucin degradation and host–microbe interactions. Elevated *Verrucomicrobiota* abundance under heat stress may reflect enhanced utilization of host-derived substrates, suggesting compromised gut barrier integrity, or altered mucosal metabolism in HS cows.

At the genus level, several core taxa, including *Faecousia*, *Cryptobacteroides*, *Alistipes*, and *Phocaeicola*, were consistently detected in both groups (Figure 1F), highlighting a shared microbial framework in the bovine hindgut. However, HT cows exhibited higher levels of *Faecousia*, *UBA737*, and *Treponema_D*, genera associated with fiber degradation, short-chain fatty acid production, and enhanced energy metabolism, whereas HS cows were enriched in unclassified genera, *HGM04593*, and *UBA4372*, taxa potentially associated with environmental stress adaptation and opportunistic colonization.

This section may be divided by subheadings. It should provide a concise and precise description of the experimental results, their interpretation, as well as the experimental conclusions that can be drawn.

### 3.3. Differential Microbial and Functional Pathway Analysis

**Figure 2:**
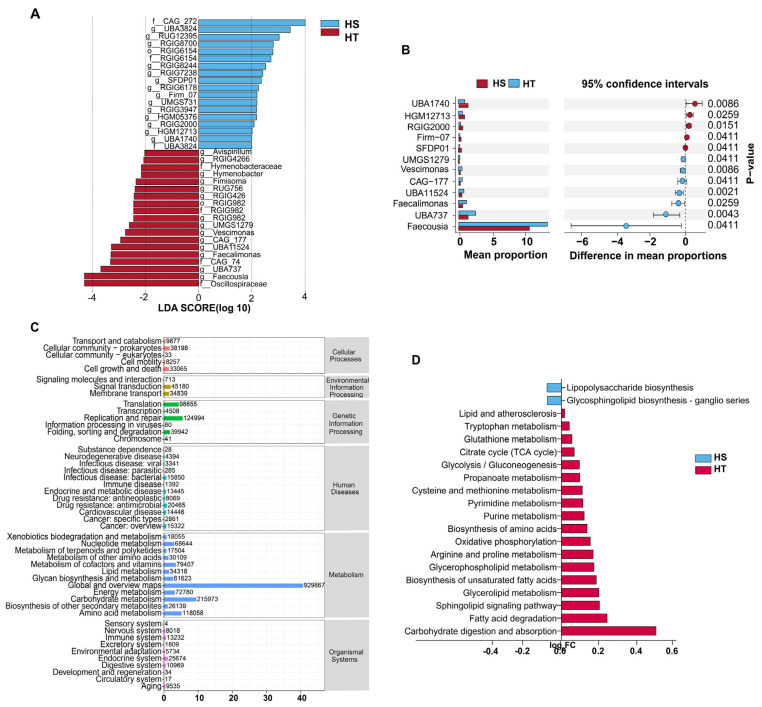
Differential microbial composition and functional pathway analysis in six heat-tolerant (HT) and six heat-sensitive (HS) cows. (**A**) Linear discriminant analysis effect size (LEfSe) identifying differential bacterial genera (LDA score >2.0). (**B**) Differential abundance analysis. The left panel shows mean relative abundances, and the right panel shows differences with 95% confidence intervals and significance levels. (**C**) KEGG level-2 classification illustrating the distribution of metagenomic functions across major metabolic categories. (**D**) Twenty heat stress-related differential pathways identified at the KEGG level-3 resolution. Bars indicate relative differences between HT and HS cows.

Differential taxonomic analysis highlighted clear distinctions in gut microbiota between HT and HS cows (Figure 2). LEfSe analysis identified 37 discriminative taxa (LDA score > 2.0), with 22 taxa enriched in HT and 15 in HS cows. In particular, HT cows showed enrichment of several *Firmicutes*-related genera, including *Faecousia*, *UBA737*, *UBA11524*, *Vescimonas*, and *CAG-448*, whereas, the HS cows harbored higher abundances of taxa such as *UBA1740*, *HGM12713*, *RGIG2000*, *SFDP01*, and *UMGS1279* (Figure 2A, Supplementary Table S2). These discriminatory taxa represent distinct microbial signatures associated with heat tolerance phenotypes.

Complementary differential abundance testing further confirmed these patterns (Figure 2B). HT cows displayed significantly higher proportions of *Faecousia* and *UBA737*, while HS cows showed greater relative abundance of *UBA1740*, *HGM12713*, and *RGIG2000*. The 95% confidence interval plots provided robust statistical support for these shifts, underscoring their potential role as group-specific microbial biomarkers.

Functional annotation of metagenomic genes against KEGG databases demonstrated that the majority of microbial functions were classified into metabolism-related pathways, including carbohydrate metabolism, amino acid metabolism, lipid metabolism, and energy metabolism, followed by genetic information processing and cellular processes (Figure 2C). This distribution reflects the fundamental roles of rumen microbiota in nutrient turnover and host energy supply.

At the KEGG level-3 resolution, multiple metabolic pathways were differentially represented between HT and HS cows (Figure 2D). The HT cows exhibited enrichment in pathways such as carbohydrate digestion and absorption, fatty acid degradation, glycerolipid metabolism, sphingolipid signaling, oxidative phosphorylation, and amino acid biosynthesis, suggesting enhanced microbial contributions to energy harvest and lipid utilization. In contrast, the HS cows showed higher abundance of pathways related to bacterial lipopolysaccharide (LPS) endotoxin biosynthesis and glycosphingolipid biosynthesis, functions often associated with cell wall remodeling, microbial stress adaptation, and a pro-inflammatory environment. Collectively, these findings indicate that the gut microbiota of HT cows is functionally skewed toward efficient energy metabolism and membrane lipid homeostasis, whereas HS cows harbor microbial communities enriched in pathways linked to stress responses and potential host inflammation.

### 3.4. Metabolomic Analysis Identifies Key Biomarkers of Heat Tolerance

**Figure 3:**
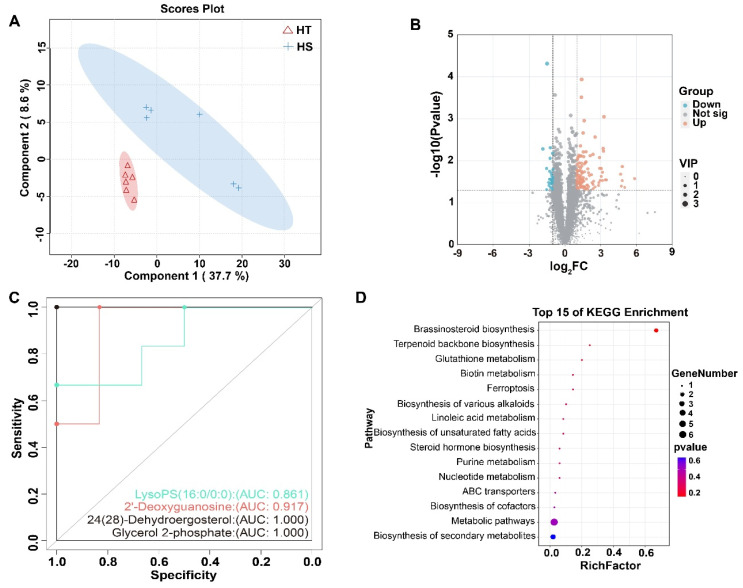
Fecal metabolomic profiling of six heat-tolerant (HT) and six heat-sensitive (HS) cows. (**A**) PLS-DA score plot showing separation of metabolic profiles between HT and HS groups. (**B**) Volcano plot of differential metabolites (criteria: |log_2_FC| ≥ 1, VIP ≥ 1, *p* < 0.05). Orange = upregulated; blue = downregulated; gray = not significant (Not sig). (**C**) Receiver operating characteristic (ROC) curve analysis of key metabolites, including LysoPS (16:0/0:0), 2′-Deoxyguanosine, 24(28)-Dehydroergosterol, and Glycerol 2-phosphate. (**D**) KEGG pathway enrichment analysis of differential metabolites visualized by bubble plot (top 15 pathways). Dot size represents the number of metabolites, and color indicates the *p*-value.

Untargeted LC–MS-based metabolomic profiling was conducted to characterize metabolic alterations between HT and HS cows (Figure 3). A total of 4747 metabolites were detected across positive and negative ion modes, providing comprehensive coverage of fecal metabolic signatures. The multivariate analysis using PLS-DA revealed a clear separation between HT and HS cows (Figure 3A), indicating distinct overall metabolic profiles between groups. Model validation supported the robustness of the separation, reflecting consistent metabolic reprogramming associated with heat tolerance status.

Volcano plot analysis further identified a panel of significantly altered metabolites (|log_2_FC| ≥ 1, VIP ≥ 1, *p* < 0.05), with 135 compounds showing differential abundance between groups (Figure 3B, Supplementary Table S3). Among these, 107 metabolites were enriched in HT cows, whereas, 25 were elevated in HS cows, suggesting distinct metabolic shifts in response to thermal stress.

To evaluate the discriminatory potential of individual metabolites, ROC analysis was performed (Figure 3C). Several metabolites demonstrated strong predictive value for heat tolerance. Glycerol 2-phosphate and 24(28)-Dehydroergosterol achieved perfect classification performance (AUC = 1.000), while 2′-Deoxyguanosine (AUC = 0.917) and LysoPS(16:0/0:0) (AUC = 0.861) also exhibited high diagnostic accuracy. These metabolites represent promising candidate biomarkers for differentiating HT and HS phenotypes.

KEGG pathway enrichment analysis of differential metabolites revealed significant involvement in lipid metabolism, nucleotide metabolism, and redox-related pathways (Figure 3D). Specifically, enriched pathways included steroid biosynthesis, purine and nucleotide metabolism, linoleic and unsaturated fatty acid metabolism, glutathione metabolism, and ferroptosis. These results highlight microbial–host metabolic interactions related to membrane integrity, energy homeostasis, and oxidative stress regulation under heat stress conditions.

### 3.5. Integrated Analysis of Metagenomics and Metabolomics

**Figure 4:**
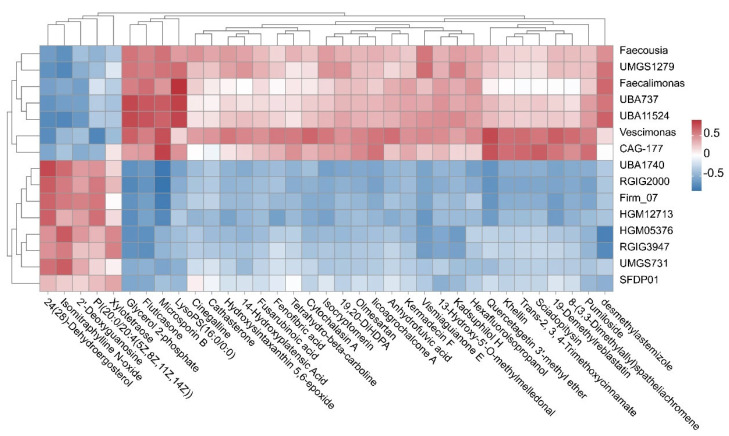
Spearman correlations between differential genera and metabolites generated from six heat-tolerant (HT) and six heat-sensitive (HS) dairy cows. The *x*-axis shows the top 15 genus-level differential taxa, while the *y*-axis shows the top 35 differential metabolites ranked by VIP values.

To elucidate potential interactions between gut microbial taxa and fecal metabolites, we performed an integrated Spearman correlation analysis combining the top 15 differential genera with the top 35 differential metabolites (|*r*| ≥ 0.6, *p* < 0.05). The resulting heatmap (Figure 4) and Supplementary Table S4 revealed 90 significant genus–metabolite associations, with several metabolites emerging as central nodes. In particular, Glycerol 2-phosphate (11 significant links), 24(28)-Dehydroergosterol (10 links), and Microsporin B (10 links) exhibited the highest degree of connectivity, followed by metabolites such as Fluticasone, Quercetagetin 3′-methyl ether, Isomitraphylline N-oxide, and desmethylastemizole.

Several robust positive correlations were identified between HT-associated genera and key metabolites. *Faecalimonas*, *UBA737*, and *UBA11524* showed strong positive associations with LysoPS (16:0/0:0) (*r* = 0.818–0.898, *p* < 0.01), while *UBA737* and *UBA11524* were also positively correlated with Glycerol 2-phosphate (*r* = 0.797–0.808, *p* < 0.01). *CAG-177* displayed significant positive correlations with Microsporin B (*r* = 0.831, *p* < 0.001) and Quercetagetin 3′-methyl ether (*r* = 0.765, *p* < 0.01). Collectively, these associations suggest that HT-enriched taxa are functionally linked to lipid remodeling, nucleotide turnover, and polyphenol metabolism.

By contrast, genera enriched in HS cows showed opposite trends. *RGIG2000*, *UBA1740*, and *Firm_07* were negatively correlated with Microsporin B (*r* = −0.793 to −0.843, *p* < 0.01), while *RGIG2000* also showed a significant negative correlation with Glycerol 2-phosphate (*r* = −0.731, *p* < 0.01). Interestingly, 24(28)-Dehydroergosterol served as a mixed hub, displaying positive correlations with HS-enriched taxa (*UBA1740* and *RGIG2000*) but negative associations with the HT-associated genus *UBA11524*. These findings suggest taxon-specific preferences for sterol-related metabolites.

Hierarchical clustering further separated the genus–metabolite relationships into two coherent modules. The first, aligned with HT cows, included *Faecalimonas*, *UBA737*, *UBA11524*, *Vescimonas*, and *CAG-177*, which clustered together with LysoPS(16:0/0:0), Glycerol 2-phosphate, and polyphenol-related metabolites. The second, aligned with HS cows, comprised *RGIG2000*, *UBA1740*, *Firm_07*, *HGM12713*, *HGM05376*, *RGIG3947*, and *UMGS731*, which correlated with metabolites indicative of xenobiotic metabolism and stress responses, such as Isomitraphylline N-oxide.

## 4. Discussion

Heat stress is a major environmental factor that disrupts physiological homeostasis in dairy cows, with one of its core mechanisms being the impairment of intestinal barrier function and consequent dysbiosis of the microbial community, leading to oxidative stress, inflammation, and metabolic disorders [26,27]. Loss of intestinal homeostasis reduces nutrient utilization efficiency and immune defense capacity, thereby exacerbating the burden of heat stress on the host [28]. Thus, elucidating the adaptive changes in gut microbiota and their metabolites is crucial for understanding the mechanisms underlying heat tolerance in dairy cows.

Metagenomic analysis revealed no significant differences in alpha diversity between HT and HS cows, whereas beta diversity clearly distinguished the two groups. In particular, PCoA showed a quantifiable separation (PC1 = 43%, PC2 = 12%) and PERMANOVA confirmed significant compositional divergence (*R*^2^ = 0.1691, *p* = 0.047). Additionally, although both groups shared a large core microbiota, HS cows harbored 3004 unique OTUs, nearly threefold more than HT cows (1098 OTUs), indicating greater instability under heat stress. Similar findings have been reported in other species and stress models. Park et al. [29] demonstrated that heat stress significantly altered the composition and functional features of the ruminal microbiota in lactating cows, with negligible effects on alpha diversity; likewise, Xie et al. [30] observed that beta diversity more effectively distinguished diarrheal phenotypes in weaned piglets, while alpha diversity differences were minimal. Together, these results highlight that microbial community structure and function, rather than diversity indices alone, provide more explanatory power for host phenotypic differences under stress conditions.

Specifically, HT cows were enriched in genera such as *Faecousia*, *UBA737*, and *Vescimonas*. Notably, *Faecousia* and *UBA737* belong to *Oscillospiraceae*, while *Vescimonas* belongs to *Ruminococcaceae*, all of which are typically associated with dietary fiber degradation and the production of SCFAs [31,32,33,34]. SCFAs not only provide important energy substrates for the host but also play essential roles in maintaining redox balance and immune regulation [35,36,37]. Thus, enrichment of these taxa in HT cows likely confers greater energetic and antioxidant support. In contrast, HS cows showed higher abundances of poorly characterized *bacteroidota*-affiliated taxa, such as the *CAG-272* and *UBA4372* lineages. Although genus-level functions remain undefined, *Bacteroidota* are known for complex polysaccharide utilization and immunomodulatory LPS biosynthesis [38,39]; given that these lineages derive from metagenome-assembled genomes without cultured representatives [40], their enrichment may indicate a carbohydrate-driven, potentially pro-inflammatory shift under heat stress. Functional annotation further confirmed this divergence. The HT cows exhibited higher gene abundances in pathways associated with energy homeostasis and antioxidant regulation, such as the carbohydrate digestion and absorption, TCA cycle, and fatty acid degradation, whereas, the HS cows were relatively enriched in pathways associated with inflammation, such as lipopolysaccharide and glycosphingolipid biosynthesis. Collectively, these findings indicate that the gut microbiota of heat-tolerant individuals contributes to enhanced energy metabolism and antioxidant defenses, while heat-sensitive individuals are more prone to inflammation-driven metabolic stress.

Metabolomic analysis provided additional insights into the distinct metabolic strategies employed by HT and HS cows under heat stress. In fecal samples from HT individuals, glycerol 2-phosphate and 24(28)-dehydroergosterol were significantly elevated. Glycerol 2-phosphate (glycerol-3-phosphate) serves as a key intermediate linking glycolysis to phospholipid metabolism, supplying substrates for membrane synthesis and remodeling, while also participating in respiratory chain electron transfer to regulate energy metabolism and redox balance. It is recognized as a critical metabolic node under high energy demand and stress conditions, helping to sustain cellular redox homeostasis and mitigate environmental challenges [41]. Similarly, 24(28)-dehydroergosterol, a derivative of ergosterol, is an essential membrane sterol that embeds into lipid bilayers to maintain membrane stability and fluidity, thereby preserving cellular integrity under external stress [42]. Ergosterol and its derivatives also possess strong antioxidant properties, enabling the scavenging of free radicals and alleviation of oxidative damage, thus enhancing cellular resilience [43]. Importantly, both metabolites achieved perfect diagnostic performance in ROC analysis (AUC = 1.000), underscoring not only their mechanistic importance in metabolic adaptation, but also their potential as robust molecular biomarkers of heat tolerance.

By contrast, fecal samples from HS cows exhibited elevated levels of LysoPS(16:0/0:0) and 2′-deoxyguanosine, suggesting a metabolic profile more strongly oriented toward inflammation and damage responses. LysoPS is a key immunomodulatory lipid that signals through G protein-coupled receptors to regulate immune cell functions, influencing both the amplification and resolution of inflammatory responses [44]. Depending on the physiological context, LysoPS can exert bidirectional effects on macrophages and other immune cells, either promoting pro-inflammatory cytokine release or facilitating resolution and tissue repair [45,46]. The elevated LysoPS levels in HS cows therefore suggest a greater susceptibility to inflammation and impaired immune homeostasis. Meanwhile, 2′-deoxyguanosine and its oxidized derivative 8-oxo-2′-dG are classical biomarkers of oxidative DNA damage and repair, with their accumulation reflecting compromised genomic stability and activation of repair processes [47,48,49]. Thus, the higher levels of 2′-deoxyguanosine in HS cows further indicate heightened vulnerability to oxidative DNA damage and repair burden under heat stress.

The integrative multi-omics analysis provided more mechanistic insights into the development of heat tolerance. Tight couplings between specific taxa and key metabolites were observed, such as the association between *UBA737* and glycerol 2-phosphate, which suggests coordinated regulation of energy metabolism and membrane synthesis, and the correlation between *Faecousia* and LysoPS, implicating microbe–metabolite interactions in modulating inflammatory signaling. These findings suggest that the advantage of heat-tolerant individuals does not arise from isolated microbial taxa or metabolites, but rather from cross-level regulatory networks integrating metabolic reprogramming with microbial functions.

Although the number of animals included in this study was limited, the selection strategy and multi-omics framework ensured the robustness of the findings. The HT and HS cows were identified through an extensive phenotypic evaluation of 120 individuals using an entropy-weighted TOPSIS model, which enabled the selection of two sharply contrasting extreme phenotypes. Extreme-phenotype designs are widely used in multi-omics studies because they enhance biological contrast and increase effect sizes, allowing reliable detection of meaningful signals even with modest sample sizes. The consistency observed across microbial, metabolic, and integrative analyses further supports the adequacy of the sample size within this experimental context.

Unlike previous studies that primarily focused on transcriptomics or proteomics [50], our study revealed that the microbial–metabolite interaction network is strongly associated with heat tolerance in dairy cows, suggesting a potential role in thermoregulatory adaptation. This integrative approach allowed us to capture multilayered mechanisms involving energy balance, membrane stability, and immune regulation with greater precision. Particularly, the identification of glycerol 2-phosphate and 24(28)-dehydroergosterol not only highlighted the metabolic advantages of heat-tolerant individuals but also revealed their potential as candidate molecular biomarkers.

## 5. Conclusions

Overall, this establishes a “microbe–metabolite interaction” framework that elucidates how coordinated microbial functions and metabolic reprogramming jointly support thermal adaptation in dairy cows. Integrative metagenomic and metabolomic analyses revealed distinct gut microbial structures and metabolic profiles between HT and HS individuals. HT cows were enriched in *Oscillospiraceae* and *Ruminococcaceae* associated with energy metabolism and antioxidant defense, and exhibited higher levels of glycerol 2-phosphate and 24(28)-dehydroergosterol—metabolites that enhance membrane stability, energy balance, and oxidative protection. In contrast, HS cows showed enrichment of inflammation-related taxa and metabolites linked to oxidative DNA damage. These findings provide molecular evidence that gut microbial–metabolite interactions are central to thermal resilience, offering valuable guidance for precision phenotyping and selective breeding of heat-tolerant dairy cattle.

## Data Availability

The original contributions presented in this study are included in the article/Supplementary Materials. Further inquiries can be directed to the corresponding author.

## Supplementary Materials

The following supporting information can be downloaded at: https://www.mdpi.com/article/10.3390/microorganisms13122829/s1, Table S1: Sequencing output and quality-control metrics for metagenomic data; Table S2: Differential microbial taxa identified by LEfSe between HT and HS cows (LDA > 2, *p* < 0.05); Table S3: Differential fecal metabolites between HT and HS cows (|log_2_FC| ≥ 1, VIP ≥ 1, *p* < 0.05); Table S4: Genus–metabolite associations from the integrative analysis (Spearman correlations; edges retained at |*r*| > 0.6 and *p* < 0.05).

## Abbreviations

The following abbreviations are used in this manuscript:

AUC	Area under the curve
HT	Heat-tolerant
HS	Heat-sensitive
KEGG	Kyoto Encyclopedia of Genes and Genomes
LDA	Linear Discriminant Analysis
LEfSe	Linear Discriminant Analysis Effect Size
ORF	Open reading frame
OTU	Operational taxonomic unit
PCA	Principal component analysis
PCoA	Principal Coordinates Analysis
PLS-DA	Partial least squares discriminant analysis
ROC	Receiver Operating Characteristic
SCFA	Short-chain fatty acid
TPM	Transcripts per million
